# Cutaneous and Mucocutaneous Leishmaniasis: Perspectives on Immunity, Virulence, and Treatment

**DOI:** 10.3390/biomedicines13123008

**Published:** 2025-12-08

**Authors:** Regina Maia de Souza, Felipe Francisco Tuon, José Angelo Lauletta Lindoso, João Vitor Matachon Viana, Isabel Aragão Maia, Raimunda Nonata Ribeiro Sampaio, Valdir Sabbaga Amato

**Affiliations:** 1 Laboratório de Parasitologia, Instituto de Medicina Tropical, Faculdade de Medicina, Universidade de São Paulo, São Paulo 05403-000, SP, Brazil; regina.maia@hc.fm.usp.br (R.M.d.S.); valdiramato@usp.br (V.S.A.); 2 Laboratório de Doenças Infecciosas e Emergentes, Pontifícia Universidade Católica Do Paraná, Curitiba 80215-901, PR, Brazil; 3 Instituto de Infectologia Emílio Ribas, São Paulo 01246-900, SP, Brazil; jose.lindoso@emilioribas.sp.gov.br (J.A.L.L.); joao.viana@emilioribas.sp.gov.br (J.V.M.V.); 4 Laboratório de Protozoologia, Instituto de Medicina Tropical, Faculdade de Medicina, Universidade de São Paulo, São Paulo 05403-000, SP, Brazil; 5 Centro Universitário Afya, Montes Claros 39408-007, MG, Brazil; belmaia.fisio@gmail.com; 6 Departamento de Infectologia e Medicina Tropical, Faculdade de Medicina, Universidade de São Paulo, São Paulo 05403-000, SP, Brazil; 7 University Hospital of Brasília, University of Brasília, Brasília 70910-900, DF, Brazil; rnrsampaio@hotmail.com

**Keywords:** *Leishmania*, leishmaniasis, parasite, lipophosphoglycan

## Abstract

Leishmaniasis, a neglected tropical disease caused by protozoa of the genus *Leishmania*, presents a wide clinical spectrum from self-healing cutaneous lesions to life-threatening visceral disease. Its epidemiology and severity vary by geography and species (Old vs. New World), vector biology, and host factors. Pathogenesis reflects a tripartite interplay among parasite, host, and sand fly saliva. Parasite virulence determinants—including lipophosphoglycan, GP63, proteophosphoglycans, and GPI-anchored antigens—facilitate complement evasion, macrophage entry, and suppression of microbicidal pathways. Innate defenses (complement, neutrophils, dendritic cells, NK cells) and PRR signaling (TLRs/NLRs) shape early outcomes, while the balance between Th1-mediated macrophage activation and Th2/regulatory responses dictates clearance versus persistence. Clinically, most infections remain cutaneous; a minority disseminate to mucosa, driven by immunopathology and species traits. Management must be individualized by *Leishmania* species, lesion burden/site, immune status, geographic region and drug availability. Local therapies (intralesional antimonials, cryo-/thermotherapy) are suitable for limited disease, whereas systemic agents (antimonials, amphotericin B, miltefosine, pentamidine, azoles) are reserved for complex, mucosal, disseminated, or immunosuppressed cases. Drug resistance—via altered uptake/efflux, metabolic rewiring, and genomic plasticity—increased toxicity and treatment failure. Targeting parasite virulence and unique metabolic pathways, improving species-specific diagnostics, and integrating host-directed strategies are priorities to shorten therapy and improve clinical outcomes.

## 1. Introduction

Leishmaniasis is a parasitic disease caused by protozoa and classified among the neglected tropical diseases (NTDs), exerting a profound impact on public health and imposing a substantial socioeconomic burden worldwide [1]. Each year, approximately 1.3 million new cases are reported globally [2]. This tropical disease presents with diverse clinical manifestations, ranging from benign, self-healing cutaneous leishmaniasis (CL) to life-threatening visceral leishmaniasis (VL), depending on the infecting *Leishmania* species [3,4,5,6,7].

Leishmaniasis is caused by protozoa of the genus *Leishmania*, which are obligate intracellular parasites of the human mononuclear phagocyte system [8]. Transmission occurs through the bite of infected phlebotomine sandflies, primarily species of the genus Phlebotomus in the Old World and Lutzomyia in the New World [9]. Host susceptibility to leishmaniasis is strongly influenced by malnutrition, immunosuppression, and genetic background [10,11].

Geographically, leishmaniases are broadly categorized into two major groups based on their distribution: (1) the Old World, encompassing regions such as the Mediterranean basin, the Middle East, the Horn of Africa, and the Indian subcontinent, where species such as *L. (L.) major*, *L. infantum*, and *L. (L.) tropica* are prevalent; and (2) the New World, primarily covering Latin America and the Caribbean, which includes species such as *L. (L.) amazonensis*, *L. (L.) chagasi*, *L. mexicana*, *L. (Viannia) naiffi*, *L. (V.) braziliensis*, and *L. (V.) guyanensis* [1,4,12,13].

Tegumentary leishmaniasis is classically represented by four clinical syndromes caused by New World *Leishmania* species. It primarily includes localized cutaneous leishmaniasis (LCL) and mucocutaneous leishmaniasis (MCL), along with less frequent manifestations such as diffuse (DCL) and disseminated CL [1]. Although typically associated with the New World, these clinical forms have also been reported in the Old World. Fewer than 5% of cases progress to mucosal disease, which is associated with severe complications and often characterized by destructive mucosal inflammation [14,15,16]. The parasite virulence, host immune response, characteristics of the sandfly vector, inoculation dose and route, and the parasite’s ability to maintain macrophages in an inert and deactivated state [10,17,18].

The pathogenesis of leishmaniasis results in a broad clinical spectrum that ranges from asymptomatic infection to chronic, debilitating disease. These outcomes are largely determined by *Leishmania* virulence mechanisms, which can be categorized as invasive/evasive, pathoantigenic, and protective [10,19]. Geographic location plays a critical role in clinical decision-making regarding therapeutic strategies. Old World species are typically associated with self-limiting cutaneous ulcers, whereas New World species often progress with greater severity and may involve mucosal tissues. Therefore, treatment must be individualized, as not all species display the same therapeutic susceptibility. Accurate species identification contributes significantly to improved clinical outcomes [1,4,12,13].

The development of novel therapeutic agents must account for the distinct pharmacokinetic profiles of the diverse clinical forms of leishmaniasis [20,21]. This complexity is further compounded by the substantial genetic variability among *Leishmania* species and the metabolic differences between promastigotes and amastigotes [1].

While intralesional therapies are considered the first-line treatment for most patients with CL [22], cases involving mucocutaneous, disseminated, or visceral disease require systemic therapeutic approaches [1]. Immunosuppression represents a major risk factor for both primary infection and reactivation of CL, underscoring the importance of tailored clinical management for patients living in or returning from endemic regions [23,24,25,26,27]. Immunosuppression—once primarily associated with HIV infection—is now increasingly prevalent worldwide, driven by advances in chronic disease management and the widespread use of immunosuppressive therapies [23,25,26,28].

The rising prevalence of immunosuppression, and the need for prolonged therapy render disease control challenging. Nevertheless, *Leishmania* virulence factors constitute promising targets for the development of novel drugs or vaccines, with the potential to shorten treatment duration and significantly improve clinical outcomes [29].

## 2. *Leishmania*

*Leishmania* exhibits a dimorphic life cycle, replicating as extracellular promastigotes within the sandfly vector and as intracellular amastigotes within mammalian hosts [6]. Numerous *Leishmania* species participate in the transmission cycle, and growing evidence indicates natural hybridization events between genetically distinct—and even phylogenetically distant—*Leishmania* species [30].

The basic cellular architecture of *Leishmania* includes the nucleus and single-copy organelles such as the mitochondrion and Golgi apparatus. Anterior to the nucleus lies the kinetoplast, a concatenated network of mitochondrial DNA physically connected to the basal body from which the flagellum emerges; this structure is likely the first part of the parasite to interact with host macrophages [31]. The glycosome and mitochondrion serve as the primary sites of energy metabolism, hosting glycolysis and the Krebs cycle, as well as oxidative phosphorylation, respectively. Several therapeutic strategies have been investigated to alleviate symptoms and reduce parasite burden. Although mitochondria-targeted approaches remain a major focus, additional promising therapeutic avenues have emerged in recent years [21]. Recent advances in leishmaniasis therapy highlight the potential of mitochondrial-targeted strategies, given the central role of the parasite’s single, unique mitochondrion in energy production, redox balance, and programmed cell death [32]. Several compounds directly disrupt mitochondrial function in *Leishmania*, including alkylphosphocholines such as miltefosine, which induce mitochondrial membrane depolarization and apoptosis-like death [33].

## 3. Pathophysiological Mechanisms

### 3.1. Sandfly Vector Effects

Over the past decade, sand fly saliva has been recognized as a critical determinant in *Leishmania* pathogenesis [10]. The saliva of phlebotomines comprise several proteins, diversified across species, genera, and even geographic regions, reflecting specific adaptations to different host populations [34]. Vasodilatory molecules such as maxadilan (*Lutzomyia longipalpis*) enhance parasite survival by increasing lesion size and duration. These proteins also inhibit dendritic cell maturation and antigen presentation by downregulating MHC-II and co-stimulatory molecules (CD80/CD86), leading to impaired IL-12 secretion—the key cytokine driving Th1 polarization. Salivary proteins also modulate host immunity by skewing the response from Th1 to Th2, promoting IL-4 and IL-6 production while suppressing TNF-α, IFN-γ, IL-12, and nitric oxide [10]. Importantly, the resulting pathology—characterized by tissue damage, inflammation, and ulceration—arises largely from the host’s dysregulated immune response rather than from direct parasite-induced cytotoxicity, underscoring the central role of host immunopathology in disease progression.

### 3.2. Relevant Virulence Factors

Virulence determinants involved in parasite invasion or immune evasion are essential for successful infection but do not directly induce host pathology [35]. These include various surface and secreted products [36]. In contrast, pathoantigenic determinants are responsible for host immunopathology and underlie most clinical manifestations. Molecules antagonistic to these determinants act as protective factors and are associated with spontaneous or treatment-induced cure [10,19].

Several *Leishmania* virulence factors contribute to parasite survival and lesion development. Among the most important is GP63, a zinc-dependent metalloprotease that facilitates macrophage invasion, degrades complement components, and modulates host signaling pathways [37]. Additionally, lipophosphoglycan (LPG) plays a key role in resistance to complement-mediated lysis, attachment to macrophages, and evasion of oxidative stress [38]. Other relevant factors include cysteine proteases (CPA and CPB), which interfere with antigen processing and promote intracellular survival, and amastins, surface glycoproteins involved in adaptation to the acidic intracellular environment [39]. Together, these virulence determinants shape parasite persistence, tissue tropism, and the severity of clinical manifestations.

*Leishmania* effectively evades reactive oxygen species (ROS) generated during macrophage oxidative bursts by modulating host antioxidant pathways and expressing its own detoxifying enzymes. Superoxide dismutase (SOD) constitutes the first line of defense, converting superoxide radicals (O_2_^−^) into molecular oxygen and hydrogen peroxide [40]. Catalases and peroxidases then degrade hydrogen peroxide into oxygen and water, neutralizing toxic peroxides. Additionally, reduced trypanothione, maintained by trypanothione reductase, functions as a potent antioxidant, further enhancing parasite survival. Trypanothione is a unique thiol-containing molecule exclusive to trypanosomatids and plays a central role in their ability to withstand oxidative stress. Unlike mammalian cells, which rely on glutathione and thioredoxin systems, *Leishmania* depends almost entirely on the trypanothione/trypanothione reductase (TR) pathway to maintain redox homeostasis [40].

#### 3.2.1. Surface Glycocalyx Factors

The parasite surface glycocalyx includes several key virulence molecules:

Kinetoplastid Membrane Protein-11 (KMP-11): A highly conserved surface protein differentially expressed in promastigote and amastigote forms. Vaccination with plasmid-encoded KMP-11 provides protection against experimental VL and partial protection against CL It has also been explored in nanoparticle delivery systems and multivalent DNA vaccine strategies [35,41].

Proteophosphoglycans (PPGs): Glycosylated polypeptides expressed in secreted and surface-bound forms. They participate in parasitophorous vacuole formation and complement activation, suggesting roles in immune evasion—although whether these reflect specific strategies or biochemical by-products remains unresolved [35].

Lipophosphoglycan (LPG): A major membrane-anchored glycoconjugate abundant in metacyclic promastigotes but scarce in amastigotes [42,43,44]. LPG exhibits genetic polymorphism and species-specific structural variations influencing virulence [45,46]. LPG deficiency in *L. major* reduces infectivity in mice without affecting replication in the vector [47,48]. LPGs from different species elicit distinct macrophage responses; more complex structures correlate with pro-inflammatory and nitric oxide-mediated effects. LPG activates Toll-like receptors 1 and 2 (TLR1/2), key components of innate immunity, contributing to pathogenesis [44,49]. Intra- and interspecific LPG and GIPL polymorphisms are associated with differential immune modulation [29,46].

Glycosylinositol Phospholipids (GIPLs): Structurally similar to LPG and protein anchors, GIPLs are inositol-containing glycolipids that tether LPG and the major surface glycoprotein GP63 to the lipid bilayer [35].

#### 3.2.2. GPI-Anchored Proteins

Among GPI-anchored proteins, leishmanolysin (GP63) and parasite surface antigen-2 (PSA-2/GP46) are particularly relevant for parasite survival and immune modulation [29]. GPI anchors themselves can act as pathogen-associated molecular patterns (PAMPs), influencing macrophage signaling, suppressing pro-inflammatory pathways, and promoting parasite persistence.

GP63: A zinc-dependent metalloprotease abundantly expressed on promastigotes of all *Leishmania* species. GP63 plays dual roles in invasion: (1) inhibiting complement-mediated lysis and (2) promoting promastigote uptake by macrophages [50]. It interacts with macrophage receptors such as Mac-1 (CD11b/CD18) and fibronectin receptors [51], facilitating internalization [52]. GP63 also interferes with key signaling pathways, including PKC and IFN-γ–mediated JAK/STAT, and transcription factors NF-κB, STAT1, and AP-1, while inducing negative regulators such as SHP-1 [37]. Given these properties, GP63 is a promising therapeutic target for new anti-leishmanial strategies.

PSA-2: Present in all *Leishmania* species except *L. braziliensis*, PSA-2 belongs to the leucine-rich repeat (LRR) protein superfamily and mediates protein–protein interactions. Structural variability includes differences in LRR number, threonine-rich regions, and GPI anchor presence. Functionally, PSA-2 supports parasite survival by inhibiting complement-mediated lysis and promoting CR3-dependent macrophage invasion [29]. It has also been implicated in antimonial drug resistance, making it a potential biomarker for identifying resistant strains and guiding therapeutic strategies [29,53].

## 4. Pathogenesis

The pathogenesis of leishmaniasis involves a complex network of interactions between multiple factors triggered by both the host’s innate and adaptive immune responses.

### 4.1. Innate Immune Response

The innate immune response relies on resident immune cells—including dendritic cells (DCs), phagocytic cells (neutrophils and macrophages), natural killer (NK) cells, and the complement system [54]. Innate sensing is mediated through pattern recognition receptors (PRRs), such as Toll-like receptors (TLRs), macrophage mannose receptors (MMRs), and NOD-like receptors (NLRs), expressed on antigen-presenting cells (APCs), which recognize pathogen-associated molecular patterns (PAMPs) from the parasite [6,44,55].

Toll-like receptors recognize parasite lipoproteins, glycolipids, and nucleic acids; for instance, TLR2 and TLR4 bind lipophosphoglycan (LPG), while TLR9 detects unmethylated CpG-rich kinetoplast DNA, promoting IL-12 and type I IFN production [44,56,57,58].

Macrophage mannose receptors (MMRs), also known as CD206, are C-type lectin receptors expressed primarily on macrophages, dendritic cells, and certain tissue-resident mononuclear phagocytes. They recognize mannose-, fucose-, and N-acetylglucosamine-rich glycans commonly found on microbial surfaces. In *Leishmania*, the parasite’s surface glycoconjugates—particularly mannose-rich glycoproteins and lipophosphoglycan (LPG)—bind efficiently to MMRs, facilitating non-opsonic phagocytosis [59]. This interaction plays a dual role. On the one hand, it promotes parasite internalization into macrophages—its primary host cell. On the other hand, MMR engagement tends to drive an anti-inflammatory signaling profile, often characterized by reduced IL-12 and nitric oxide production and increased IL-10, creating a permissive environment for early parasite survival. Because MMR uptake bypasses strong microbicidal activation pathways, *Leishmania* exploits this receptor as an immune-evasion mechanism [60].

#### 4.1.1. Dendritic Cells and Inflammatory Monocytes

Within the first hours of infection, some DCs and monocytes are directly infected by *Leishmania*. Early recruitment of inflammatory monocytes depends on CCL2 (MCP-1, also known as Monocyte Chemoattractant Protein-1), produced at the infection site upon activation by platelet-derived growth factor. Monocyte infection differs markedly from macrophage infection: monocytes mount a strong respiratory burst, contributing to early parasite control, whereas macrophages require IFN-γ activation to effectively kill intracellular parasites [54].

#### 4.1.2. Phagocytic Cells: Neutrophils and Macrophages

Neutrophils are the first cells recruited to the site of *Leishmania* infection, where they phagocytose promastigotes and release microbicidal factors such as nitric oxide, elastase, platelet-activating factor, and neutrophil extracellular traps (NETs). By contrast, macrophages, while involved in parasite clearance, also serve as the principal intracellular niche for parasite survival. *Leishmania* exploits surface molecules to enter macrophages through CR3-mediated “silent entry,” thereby evading protective immune responses [61].

#### 4.1.3. Natural Killer Cells

In humans, reduced NK cell numbers are associated with disease progression, while their presence in healing lesions suggests a protective role. NK cells exert direct cytotoxic activity and produce IFN-γ, which induces iNOS expression in infected macrophages. Early in infection, NK cells are recruited after neutrophils and serve as an initial source of IFN-γ, promoting Th1 differentiation and limiting parasite dissemination [61].

#### 4.1.4. Complement System

The complement system plays a critical role in eliminating *Leishmania* promastigotes immediately after inoculation. Activation upon contact with serum leads to lysis of more than 90% of parasites at the inoculation site [35]. Susceptibility to complement-mediated lysis depends on serum complement concentration and varies between species: cutaneous strains are generally more susceptible, likely due to differences in surface antigen expression [35].

#### 4.1.5. Toll-like and NOD-like Receptors

TLRs represent one of the host’s first lines of defense against *Leishmania* spp. Upon PAMP recognition, TLR signaling activates NF-κB, which translocate to the nucleus and induces pro-inflammatory cytokine transcription. *Leishmania* has evolved mechanisms to modulate TLR4 signaling, facilitating infection. TLR9, expressed mainly in macrophages, is linked to granuloma formation [62]. In tegumentary leishmaniasis caused by *L. braziliensis*, TLR2 is the most frequently expressed receptor during active disease, particularly on macrophages, although expression does not directly correlate with cytokine levels.

NLRs recognize molecular patterns in the cytoplasm and contribute to immune regulation. Their role in *Leishmania* infection remains incompletely understood, with divergent study results. Known NLR functions include regulation of MHC expression and inflammasome activation (NLRP1b, NLRP3, NLRC4), promoting the production of IL-1β and IL-18.

#### 4.1.6. Inflammasome and IL-1 Axis, and Th17/TNF Pathways

Inflammasomes are critical regulators of immunity in *Leishmania* infection. Their activation drives the release of IL-1β and IL-18. IL-1β via NLRP3 contributes to nitric-oxide mediated resistance in most models but promotes susceptibility in *L. (L.) major* infection. In contrast, IL-18 favors a Th2 response and susceptibility in infections with *L. (L.) mexicana* and *L. (L.) amazonensis* [63]. Activation of the NLRP3 inflammasome and IL-1β production are associated with cytotoxicity and tissue damage in patients with LCL caused by *L. (V.) braziliensis* [64].

Studies show a predominance of Th1 pro-inflammatory cytokines prior to CL treatment, followed by a significant post-therapy reduction in TNF-α and IFN-γ [65]. Recently, the Th1/Th2 paradigm was reevaluated with the identification of a new lymphocyte subset: Th17 cells, characterized by IL-17 production, differentiate in the presence of IL-6 and TGF-β and rely on IL-23 for survival. They have a pro-inflammatory role, recruiting neutrophils and macrophages, and studies in leishmaniasis show their presence is associated with more severe lesions [66]. IL-17–producing cells are associated with the intensity of the inflammatory infiltrate [65].

#### 4.1.7. Human Microbiome

The skin microbiome contributes to homeostatic mechanisms that reinforce the skin barrier function. However, when this barrier is disrupted as in *Leishmania* infection the commensal microbiota becomes disturbed, exposing the underlying tissues to invasion by microorganisms.

*Leishmania braziliensis* infection leads to intense skin inflammation with variable clinical outcomes. In 62 patients, high bacterial burden and *Staphylococcus* spp. dominated microbiomes were linked to delayed healing and enhanced IL-1 driven inflammation. Dual RNA-seq revealed that elevated *S. aureus* transcripts correlated with increased IL-1β expression and worse prognosis. In mice, IL-1β neutralization reduced pathology, indicating that the skin microbiota modulates cutaneous leishmaniasis outcomes and represents a potential therapeutic target [67].

In animal model, *L. major* can modulate the host gut microbiota, and this interaction appears to differ according to host susceptibility. Infection with *L. major* induced pronounced alterations in the ileum microbiota, whereas the colon community remained comparatively stable. These findings provide important insight into how *Leishmania* infection and host genetic background shape gut microbial composition and metabolic potential, contributing to the understanding of host–parasite interactions [68].

## 5. Cellular Immune Response

Once the inflammatory process is established, characterized by the recruitment of macrophages and granuloma formation, a robust cell-mediated immune response is initiated. CD4^+^ T cells are the primary source of IFN-γ, which activates macrophages to eliminate intracellular parasites. The clinical outcome is determined largely by the balance between Th1 and Th2 T helper cell responses. In addition, the characterization of programmed cell death pathways in *Leishmania* is critical for efficient amastigote clearance [69].

A Th1 response, mediated by IFN-γ, TNF, and IL-12, is associated with macrophage activation, parasite killing, and resistance to disease. In contrast, the Th2 response is defined by downregulation of pro-inflammatory cytokines and increased production of IL-4, IL-10, IL-13, and TGF-β, leading to macrophage deactivation and reduced production of protective cytokines. While this response may limit tissue damage, it simultaneously facilitates the persistence of intracellular infection. A dominant Th2 profile is therefore associated with susceptibility and disease progression [10,14,15,70].

Studies of cellular immune responses in human leishmaniasis have been largely descriptive. This is mainly due to challenges in precisely defining protective versus immunopathological mechanisms, the lack of longitudinal investigations, and the substantial genetic heterogeneity of both human hosts and *Leishmania* species [10].

## 6. Role of Cytokines in Disease Progression

**Figure 1:**
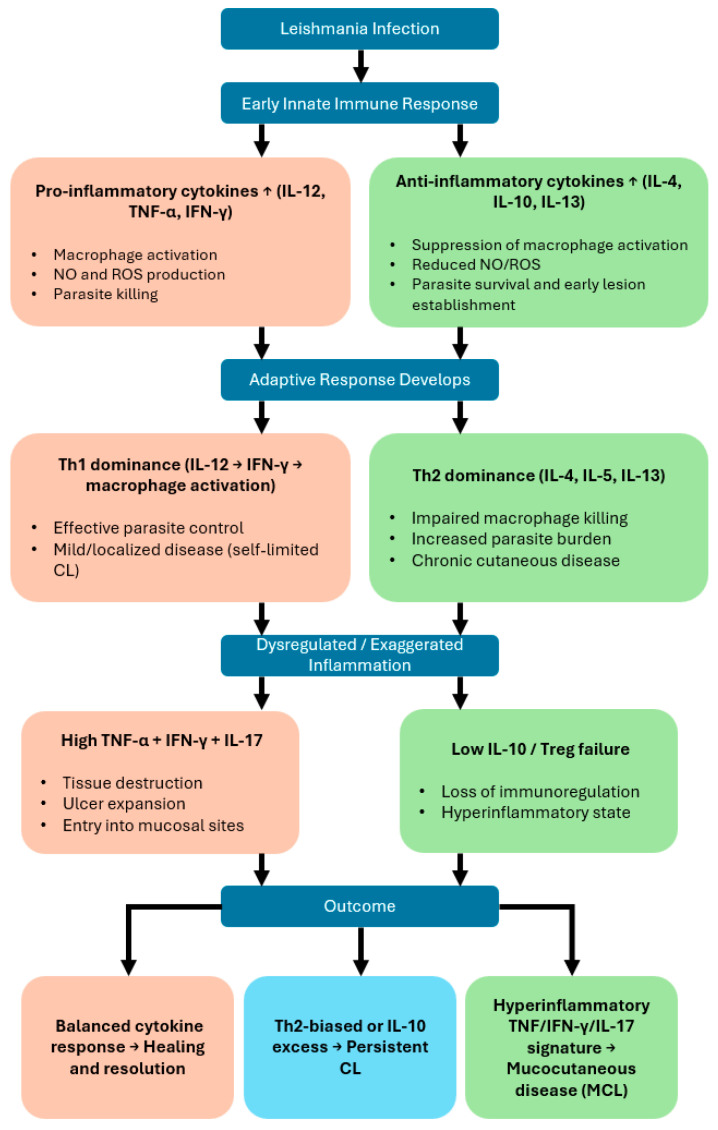
Cytokine-driven immunological pathways determining clinical outcomes in cutaneous and mucocutaneous leishmaniasis. This flowchart illustrates how cytokine responses shape the progression and clinical manifestations of *Leishmania* infection.

The activation of permissive host macrophages into leishmanicidal effector cells during *Leishmania* infection relies on cytokines, particularly IFN-γ, which is produced in large quantities during the early phase of disease, as demonstrated in situ in lesion biopsies [10]. This cytokine is secreted by multiple immune cell populations, including natural killer (NK) cells, CD4^+^ and CD8^+^ T lymphocytes, and specific NKT cell subsets (PMID: 35769465). However, IFN-γ production can be downregulated by elevated levels of interleukin-10 (IL-10), which may account for a transient period of increased local parasite replication [10] (Figure 1).

## 7. Infection

### 7.1. Pathogenesis and Clinical Manifestations

Infection begins when an infected female sand fly vector inoculates metacyclic promastigotes into the host’s skin during a blood meal. Saliva rich in vasodilatory and anticoagulant molecules with chemotactic activity enhances parasite survival by attracting phagocytic cells [40,52]. Among the *Leishmania* developmental stages, metacyclic promastigotes express the highest levels of protein kinases and GP63, which phosphorylate complement proteins C3, C5, and C9, inactivating both the classical and alternative complement pathways [71].

Immediately after inoculation, neutrophils are rapidly recruited to the site of infection, where they adhere to the endothelium and exert multiple effector functions, including phagocytosis and the formation of NETs, which release DNA-associated microbicidal agents [8,72,73]. Neutrophils are short-lived and act as “Trojan horses,” providing an intermediate niche that facilitates parasite transfer to macrophages and allows early immune evasion [74,75].

During innate immunity, TLR signaling represents a key first-line defense mechanism against *Leishmania*, recognizing molecules such as GP63 and LPG on dendritic cells, macrophages, neutrophils, and T and B lymphocytes [44,55]. Inside macrophages, GP63 degrades extracellular matrix components (e.g., collagen, fibronectin), modulates host signaling pathways, and cleaves C3 to C3bi, facilitating parasite entry through CR3-mediated phagocytosis [29,76]. LPG also confers resistance to complement lysis, suppresses oxidative burst, inhibits inflammatory signaling, and interferes with NK T cell recognition of infected macrophages [61].

Biochemical modifications during metacyclogenesis further enhance promastigote resistance to complement and innate killing mechanisms. *Leishmania* interferes with host signal transduction pathways, altering the balance between macrophage microbicidal and suppressive functions, favoring intracellular persistence. Once internalized, promastigotes transform into amastigotes within acidic phagolysosomes, where they replicate, resist oxidative stress, and propagate to new host cells.

### 7.2. Clinical Manifestations

*Leishmania* infection may remain asymptomatic—approximately 70% of exposed individuals control the infection without clinical disease [77,78]. Symptomatic disease most commonly manifests as cutaneous or mucocutaneous leishmaniasis, with rarer presentations including diffuse and disseminated forms [14,15,79]. In endemic settings, coinfections may mimic clinical lesions, leading to diagnostic delays and mismanagement [1]. The incubation period ranges from 15 to 60 days, with lesions typically evolving over weeks to months, progressing from papules to nodules and then to shallow ulcers with raised edges, usually painless and 0.5–3.0 cm in diameter [10,14,15].

#### 7.2.1. Localized Cutaneous Leishmaniasis

LCL reflects a dynamic balance between pro-inflammatory (Th1-type: IFN-γ, IL-2, TNF-α) and regulatory (Th2-type: IL-4, IL-5, IL-10, IL-13, TGF-β) cytokines, modulating macrophage activity and effector responses [80,81,82,83,84,85]. Interestingly, patients with low baseline IFN-γ levels may respond more favorably to pentavalent antimonials, which disrupt DNA replication, fatty acid oxidation, ADP phosphorylation, and glycolysis [10,21].

#### 7.2.2. Mucocutaneous Leishmaniasis

MCL results from lymphatic or hematogenous dissemination of parasites to mucosal tissues, especially in the nasal and oral cavities, causing severe inflammatory destruction of cartilage and bone, leading to respiratory and nutritional compromise [10,14,15]. Mucosal lesions may appear years or even decades after the initial cutaneous lesion, with 46% of cases within 2 years and 50% within 5 years [69,86]. Between 1% and 10% of CL cases progress to MCL within 1–5 years, though rates up to 25% have been documented [87].

Patients with MCL exhibit a mixed Th1/Th2 immune profile, with elevated IFN-γ and TNF-α production that contributes to tissue destruction rather than parasite clearance. Low IL-10 levels may exacerbate inflammation. MCL lesions show a high density of activated T cells and cytotoxic CD8^+^ lymphocytes, with high local IFN-γ, TNF-α, and IL-4, key immunopathological markers [82,88]. Genetic susceptibility also influences outcomes; for example, IL6-174 G/C promoter polymorphisms have been associated with mucosal disease, low IL-6 production, more severe inflammation, treatment failure, and relapse [89,90].

#### 7.2.3. Diffuse and Disseminated Cutaneous Leishmaniasis

Diffuse CL is characterized by T-cell anergy, massive parasite burden, poor or absent LST (*Leishmania* skin test) reactivity, and nodular lesions resembling lepromatous leprosy. Disseminated CL, in contrast, presents with necrotic ulcers, strong LST reactivity, fewer parasites, and favorable treatment outcomes [1,79]. Disseminated and mucosal involvement are common. These immunological extremes mirror the leprosy spectrum model of host response [1].

HIV co-infection significantly increases recurrence and treatment failure risk. Conversely, CL can impair innate immunity, accelerating HIV progression. Beyond HIV, other causes of immunosuppression—including transplantation, rheumatologic disease, hematologic malignancies, and cancer—also alter clinical presentation [26].

### 7.3. Immunosuppression, Persistence, and Relapse

Although the classical Th1/Th2 dichotomy provides a useful framework, host responses are more complex [10,91]. *Leishmania* can persist in host cells by inducing immunosuppression and altering chemokine networks [35,40]. In addition to HIV, immunosuppression related to transplant, autoimmune diseases, or cancer is an important risk factor [28].

Parasites may persist at low levels even after clinical resolution, controlled but not eliminated by cell-mediated immunity. Regulatory T cell induction is thought to dampen effector responses, permitting long-term parasite survival [54,71,92]. Inhibition of NOS2 during immunosuppression may reactivate latent infections, while high NO levels correlate with parasite control [93].

Importantly, *Leishmania* persistence and drug resistance involve distinct mechanisms. Persistent forms survive by increasing tolerance and entering dormancy without genetic resistance. Once drug pressure is removed, these parasites can resume proliferation while remaining susceptible to treatment [94].

Relapse in cutaneous and mucocutaneous leishmaniasis is a multifactorial phenomenon influenced by parasite, host, and treatment-related determinants [15,28]. Parasite persistence after clinical cure is a well-recognized driver of relapse, supported by evidence that *Leishmania* can remain in quiescent niches such as macrophages, fibroblasts, and peri-lesional tissues despite adequate therapy [95]. Host immunological factors also play a central role: inadequate Th1 responses, impaired IFN-γ production, and dysfunctional macrophage activation may limit the ability to fully eliminate residual parasites [41,96]. In MCL, exaggerated and dysregulated inflammatory responses paradoxically coexist with an inability to sterilize infection, further predisposing to recurrence [87]. Clinical variables—including lesion size, chronicity, inadequate wound healing, treatment delays, and coinfections—also contribute to relapse risk. Additionally, therapeutic aspects such as suboptimal drug exposure, poor adherence, and variability in pharmacokinetics/pharmacodynamics can result in insufficient parasite clearance, favoring disease recurrence. These combined factors highlight the importance of integrating clinical monitoring with immunological and therapeutic considerations to reduce relapse rates in CL and MCL.

## 8. Main Metabolic Pathways

Several *Leishmania* metabolic pathways contribute to parasite growth, differentiation, and survival within the host.

### 8.1. Purine Salvage Pathway

Unlike mammals, *Leishmania* lacks de novo purine biosynthesis and depends entirely on purine salvage. The parasite recycles adenine, guanine, hypoxanthine, and xanthine derived from host nucleotide degradation. Multiple nucleoside hydrolases catalyze the cleavage of nucleosides, nucleotides, and nucleic acids to generate purine bases that enter the parasite’s purine pools [97]. This pathway represents an attractive therapeutic target because of its essentiality and absence in humans.

### 8.2. Glycolysis Pathway

Glycolysis is a major energy source for promastigote forms, providing ATP necessary for parasite proliferation and survival within the vector and early in mammalian hosts [97].

### 8.3. GPI Biosynthesis Pathway

The glycosylphosphatidylinositol (GPI) biosynthesis pathway involves the sequential addition of sugars and ethanolamine to phosphatidylinositol (PI). It anchors key virulence factors to the parasite surface, including lipophosphoglycan (LPG), glycoinositolphospholipids (GIPLs), glycoprotein 63 (GP63), and proteophosphoglycan (PPG). Secreted phosphoglycan (PG)-containing proteins such as secreted PPG (sPPG) and secreted acid phosphatase (sAP) also derive from this pathway [97,98]. Given their role in parasite–host interactions, components of this pathway are promising drug targets.

### 8.4. Redox Metabolism: TSH2 Pathway

Resistance to oxidative stress is essential for parasite survival in the host. In trypanosomatids, trypanothione (TSH2), a unique thiol-based redox metabolite, detoxifies peroxides and supports critical cellular processes [99]. Since humans lack this pathway, its enzymes offer highly selective targets for antiparasitic drug development [100].

### 8.5. Sterol Biosynthesis Pathway

Sterols are crucial components of eukaryotic membranes, regulating fluidity and permeability and serving as key precursors and regulators in cell cycle and developmental processes [32]. In trypanosomatids, sterol biosynthesis begins with squalene epoxidation, forming squalene epoxide, which is converted to lanosterol and then to ergosterol—unlike mammals, where cholesterol is the final product [101].

Statins inhibit the host HMG-CoA reductase (HMGR), a rate-limiting enzyme in cholesterol biosynthesis. Because *Leishmania* infection outcomes are influenced by host cholesterol levels, pharmacological modulation of this pathway may offer adjunctive therapeutic strategies [102].

## 9. Treatment

Therapeutic decisions must be individualized, taking into account the *Leishmania* species, number of lesions, anatomic sites involved, and the therapeutic options available at the point of care [4,12,13,103,104,105]. Systemic therapy is primarily indicated for complex disease—e.g., multiple lesions, diameters of >5 cm, or mucosal involvement—and should also be considered for species at higher risk of mucosal dissemination, notably members of the subgenus *Viannia* (especially *L. (V.) braziliensis* and *L. (V.) guyanensis*), as a preventive strategy. Parenteral therapy is further recommended for immunosuppressed patients (including those living with HIV) and for individuals who have failed or have contraindications to local therapy [12,13,103,104].

Local therapies are recognized and recommended by WHO/PAHO as alternatives to systemic treatment when patients have up to four exclusively cutaneous lesions <4 cm in diameter that do not involve the face or joints. Benefits include lower toxicity and cost compared with systemic regimens [106]. Most drugs used against leishmaniasis were developed for other indications, and their anti-*Leishmania* mechanisms are not always fully understood. Resistance is increasingly reported; accordingly, selecting an appropriate agent and regimen requires understanding each therapy’s mechanism of action.

### 9.1. Pentavalent Antimonials (SbV)

Agents: Sodium stibogluconate (SSG, Pentostam^®^) and meglumine antimoniate (MA, Glucantime^®^).

Mechanisms: Reduction of the prodrug (SbV) to trivalent antimony (SbIII) with macrophage activation; interference with parasite energy metabolism via inhibition of glycolysis and fatty-acid β-oxidation; induction of reactive oxygen species; and inhibition of thiol-dependent metalloproteins such as trypanothione reductase, weakening antioxidant defenses [107,108,109].

Systemic dosing: Typical IV regimens: 20 mg/kg/day for 10 days for *L. infantum*; 20 days for New World CL; up to 30 days for mucosal disease. Major adverse events include cardiotoxicity (QT prolongation/arrhythmia), pancreatitis, and hepatotoxicity [22,110].

Efficacy and geography/species effects: Responses vary by species and region. For *L. (V.) braziliensis* treated for 20 days, reported efficacy was ~69.6% in Peru, 50.8% in Brazil, and 96.0% in Guatemala. Courses of 28–30 days yield ~75% cure in MCL, with worse outcomes in severe disease. Marked antimonial resistance (>60% failure) has been reported in parts of the Indian subcontinent [103].

Resistance: Decreased expression of the aquaglyceroporin transporter (reducing SbIII entry), overexpression of efflux pumps, and related mechanisms [111,112,113].

Intralesional therapy: Direct infiltration achieves high local drug levels while minimizing systemic exposure—appropriate for small, localized, non-mucosal lesions. Volume generally ~1–5 mL per session; repeat every 3–7 days until complete re-epithelialization. Local reactions include pain, edema, erythema, and pruritus. Reported cure rates: ~83% with SSG and ~68% with MA in Old World CL; ~61–82% in New World CL. Combination with cryotherapy can improve outcomes [110,114].

### 9.2. Amphotericin B

Mechanism: Binds ergosterol-like membrane sterols, forms pores, and increases permeability, leading to osmotic lysis [105,115]. Resistance—though uncommon—has been associated with alterations in membrane sterol composition that reduce drug affinity [116,117].

Dosing: Deoxycholate: 0.5–1.0 mg/kg IV daily or on alternate days to a cumulative 20–45 mg/kg for MCL. Liposomal (preferred due to lower nephrotoxicity): 3 mg/kg/day IV to a cumulative 20–60 mg/kg. A Brazilian series reported 93.1% efficacy for MCL due to *L. braziliensis* with a mean cumulative dose of 32.5 mg/kg of liposomal amphotericin B [115,118].

### 9.3. Miltefosine

Oral miltefosine, originally developed as an antineoplastic, has antileishmanial activity by disrupting lipid metabolism via inhibition of phospholipid and sterol biosynthesis—processes essential for parasite membrane biogenesis [33,119].

It is considered second-line in some settings after antimonial failure or resistance, achieving ~80–90% parasitological improvement within two months. For CL due to *L. (V.) guyanensis* or *L. (V.) panamensis*, miltefosine can be first-line at 2.5 mg/kg/day (maximum 150 mg/day) for 28 days. Activity against *L. infantum* and *L. (V.) braziliensis* is variable and may be compromised by emerging resistance [105,120,121]. Miltefosine is teratogenic and contraindicated in women of childbearing potential unless strict contraception is in place. When feasible, specialist gynecology/obstetrics evaluation is recommended, with two contraceptive methods during therapy and for three months after completion.

Resistance mechanisms: Drug entry depends on the aminophospholipid translocator; mutations/deletions in this transporter impair uptake. Efflux pump overexpression and structural alterations in phospholipid/sterol biosynthesis further reduce efficacy [119,122].

### 9.4. Pentamidine

Pentamidine (notably the isethionate salt) is indicated for CL due to *L. (V.) guyanensis*—prevalent in the Brazilian Amazon and French Guiana—and may be used when SbV is contraindicated or not tolerated. Results for other species are heterogeneous. Cure rates up to ~96% for *L. (V.) guyanensis* have been achieved with three weekly intramuscular doses of 7 mg/kg. Routes include IM, IV, or intralesional administration, the latter reserved for single, small lesions [123,124,125,126].

Resistance mechanisms: Pentamidine accumulates in the kinetoplast and inhibits topoisomerase IB, blocking kDNA replication and leading to parasite death. Resistance is linked to reduced mitochondrial membrane potential (lower intrakinetoplastic accumulation), altered membrane transporters, and kDNA minicircle changes [127,128,129].

### 9.5. Azoles

Imidazoles and triazoles deplete ergosterol and block its synthesis, leading to accumulation of toxic sterol intermediates and impaired membrane function. Ketoconazole, itraconazole, and fluconazole are orally administered and generally well tolerated, but CL outcomes vary globally [130,131,132,133]. The cure rate is ~64% overall, with higher rates for *L. mexicana* (89%), *L. infantum* (88%), and *L. donovani* (80%), and lower for *L. major* (53%), *L. (V.) braziliensis* (49%), and *L. tropica* (15%) [130,132]. The suggested regimens include fluconazole 200–400 mg/day for 6 weeks; ketoconazole 600 mg/day for 28 days; or itraconazole 200 mg/day for 42–56 days [130].

Resistance mechanisms: *Leishmania* resistance to imidazoles and triazoles arises mainly from mutations or overexpression of the target enzyme CYP51, remodeling of the sterol-biosynthesis pathway, and increased drug efflux via ABC transporters.

### 9.6. Paromomycin

Paromomycin, an aminoglycoside, can be used topically for selected CL cases—preferably ≤10 small, superficial lesions not involving cosmetically/functionally sensitive sites (face or joints) and caused by susceptible species such as *L. major* and *L. panamensis* [134,135,136]. The therapeutic regimen consists of daily application of 15% aquaphilic paromomycin for 20–30 days with a cure rate of ~75–82%. Adverse effects are mild and localized (pruritus, pain, dermatitis) [134,135].

Resistance mechanisms: Paromomycin binds cytosolic ribosomes, causing mRNA misreading and translational arrest. Resistance is associated with altered membrane permeability/fluidity that diminishes intracellular accumulation and reduces initial drug binding at the parasite surface [137,138].

### 9.7. Thermotherapy

Thermotherapy exploits the thermosensitivity of amastigotes and is theoretically applicable across *Leishmania* species. The standard method uses radiofrequency-generated heat applied locally at 50 °C for 30 s (under local anesthesia after skin antisepsis), covering the entire lesion and ~2 mm margins. Expected adverse effects include pain, pruritus, and occasional second-degree burns [12,103,139].

Mechanisms and efficacy. Thermal cytotoxicity directly kills amastigotes and may secondarily modulate cytokine responses (e.g., IFN-γ, IL-5, TNF-α), aiding lesion resolution [140,141]. Reported cure rates range from ~54% to 81%, with better outcomes for small lesions (<25 mm), few in number (≤1–2), and not involving face or joints. Thermotherapy should be considered when antimonials are contraindicated or have failed [139,140,141,142].

### 9.8. Cryotherapy

Cryotherapy applies a cryogenic agent (e.g., liquid nitrogen or carbon dioxide) directly to the lesion, inducing tissue destruction by freezing; infected cells undergo local necrosis, eliminating amastigotes. Sessions last 15–30 s, covering the entire lesion and ~1 mm margins, and may be repeated weekly or every three weeks based on clinical response [105,142,143].

Efficacy and safety: Cure rates up to ~88% after 1–4 sessions, especially for lesions <30 mm and ≤3 months’ duration. Adverse effects include vesiculation, erythema, edema, and local pain; pigmentary changes and hypertrophic/keloid scarring are uncommon. Cryotherapy is considered safe during pregnancy and lactation [142,144]. It can be used alone or combined with intralesional antimonials to enhance cure and is a reasonable option for residual lesions after systemic therapy [144,145,146].

### 9.9. Emerging Immunotherapeutic Approaches

Blocking PD-1 or its ligand PD-L1 restores T cell function in different types of cancer, which is associated with a better disease prognosis. A study provided evidence that the PD-1/PD-L1 pathway plays a key regulatory role in the immune response to CL, provides a delicate fine-tuning of the immune response, which ensures an optimal anti-parasitic immune response without overwhelming inflammation [147].

The outcome of CL is largely determined by the balance of antigen-specific T cell responses: IFN-γ production promotes parasite control, whereas excessive TNF-α and nonspecific cytotoxicity contribute to cutaneous inflammation and lesion pathology. In addition, the lack of inhibitory regulatory mechanisms exacerbates tissue damage. During prolonged antigen exposure or chronic inflammation, T cells progressively lose effector function due to the expression of inhibitory checkpoint receptors, including programmed death 1 (PD-1), T cell immunoglobulin-3 (TIM-3), Cytotoxic T-lymphocyte-associated protein 4 (CTLA-4), Lymphocyte activation gene-3 (LAG-3), and T cell immunoglobulin and ITIM domain (TIGIT) [148].

Nutritional deficiencies, including protein-calorie malnutrition, contribute to clinical worsening and reduced treatment efficacy in leishmaniasis. Zinc has emerged as an important modulator of the immune response due to its antioxidant and anti-inflammatory properties, acting as an intracellular signaling factor for immune cells [149]. Studies report low plasma zinc levels in patients with active disease and elevated copper levels. An increased copper/zinc ratio has been linked to impaired cellular immunity and enhanced humoral responses [150]. In humans, severe malnutrition was associated with at least a four-fold higher risk of developing difficult-to-manage mucosal lesions [151].

## 10. Mechanisms of Drug Resistance in *Leishmania* spp.

Classically, resistance arises from genetic alterations that reduce parasite susceptibility to a given drug [152]. Host-related factors also contribute—immune dysfunction, inappropriate antimicrobial use during medical care, and environmental dissemination—all of which can select for resistant parasites. Clinically, drug resistance drives treatment failure and relapse, facilitates transmission, prolongs hospital stays, and increases healthcare costs. Notably, how genomic alterations interface with translational control—and how these layers jointly shape resistance phenotypes—remains incompletely understood (Table 1).

Metabolomic and transcriptomic studies indicate that resistant parasites display altered and differentially expressed metabolites linked to lipid, energy, and amino-acid metabolism. Among these, lipid remodeling and broader rewiring of lipid metabolism are the best characterized to date. During mammalian infection, *Leishmania* encounters environmental stresses that trigger dynamic changes in gene expression: although global translation decreases during amastigote differentiation, select transcripts are preferentially upregulated. Because gene expression is governed predominantly at the translational and post-transcriptional levels, RNA-binding proteins (RBPs) assume central regulatory roles and can buffer the absence of classical transcriptional control [94].

At the effector level, acquired resistance commonly involves proteins that degrade or neutralize drugs, diminish uptake, or enhance export via membrane transporters [153]. Calcium-dependent protein kinase 1 (CDPK1) and ATP-binding cassette (ABC) transporters are of particular interest for drug development; translational reprogramming and metabolic shifts further enhance stress tolerance, oxidative-stress management, and membrane integrity in resistant parasites [94]. Overexpression of ABC transporters mediating ATP-dependent transmembrane flux can promote vesicular sequestration and efflux; PRP1 (a pentamidine resistance protein) has been specifically linked to pentamidine resistance [94,152].

Despite variable efficacy across species and geographies—and increasing reports of failure—antimonials remain first-line therapy for many forms of leishmaniasis, reflecting, in part, intrinsic interspecific differences in susceptibility [154]. Paromomycin acts primarily at the mitochondrion, inducing respiratory dysfunction and inhibiting protein synthesis; resistance correlates with reduced uptake and impaired initial binding at the parasite surface [154].

The *Leishmania* genome is highly plastic. Under stress, parasites deploy aneuploidy, gene amplification, and gene deletion to modulate gene dosage and expression, thereby impacting targets, transporters, and drug-inactivating enzymes [155,156,157]. Single-nucleotide polymorphisms (SNPs) that alter protein structure/function also contribute [94,158]. Polyploidy and chromosomal copy-number gains are frequent in drug-resistant isolates; genes such as MRPA (Multidrug Resistance Protein A), APX (Ascorbate Peroxidase), and G6PDH have been repeatedly implicated in antimony resistance through dosage effects [94,157]. Aneuploidy-associated changes have been observed in paromomycin-resistant lines [159]. Deletions or reduced expression of AQP1, the aquaglyceroporin that mediates Sb(III) uptake, underpin resistance to both antimonials and pentamidine [94,160,161,162]. By contrast, copy-number variation and aneuploidy did not associate with amphotericin B resistance in at least one study [163].

Combination therapy is a rational strategy to curb resistance emergence. For miltefosine (MLT), mutations in the MLT transporter and deletion of the MLT-sensitive locus have been described in multiple *Leishmania* species and have been associated with cross-resistance to amphotericin B [164]. More broadly, integrated multi-omics approaches—combining proteomics, genomics, and transcriptomics—are considered more promising for anti-leishmanial drug discovery than target identification in isolation [165]. In parallel, curated biomarker panels should guide diagnosis and therapeutic decision-making for resistant species at country/region level to optimize outcomes [7].

**Table 1:** Major mechanisms of drug resistance in *Leishmania* spp. by drug class.

Drug Class/Agent	Molecular Mechanism	Genes/Proteins Involved	Functional Consequence	Strategy to Overcome
Antimonials (SbV/SbIII) [166]	Decreased Sb(III) uptake and increased efflux; enhanced thiol-based detoxification	AQP1, MRPA, GSH1, TRYR, ABC transporters	Reduced intracellular accumulation and neutralization of reactive oxygen species	Combination with efflux pump inhibitors; liposomal formulations; monitoring of AQP1 expression
Amphotericin B [167]	Altered sterol composition of the plasma membrane	ERG3, ERG6, ERG2, ERG11	Decreased drug binding affinity and membrane permeability	Combination with miltefosine; optimized cumulative liposomal dosing
Miltefosine [121,168]	Reduced uptake and increased efflux; deletion of the miltefosine-sensitive locus	LdMT, LdRos3, ABC transporters, MTT	Therapeutic failure and cross-resistance to amphotericin B	Pre-therapy susceptibility testing; avoidance of prolonged monotherapy
Pentamidine [169]	Reduced mitochondrial membrane potential; mutations in transporters	PRP1, AQP1, kDNA minicircle alterations	Decreased intramitochondrial accumulation and loss of cytotoxicity	Alternating or combined therapy with antimonials or amphotericin B
Paromomycin [170]	Decreased membrane permeability and intracellular accumulation; ribosomal alteration	Membrane-associated genes, EF1α, RPS6	Attenuated inhibition of protein synthesis	Liposomal formulations; combination therapy
Azoles (ketoconazole, fluconazole, itraconazole) [101]	Mutation or overexpression of enzymes in the ergosterol biosynthesis pathway	CYP51, ERG11, ERG6	Accumulation of non-lethal sterol intermediates	Combination with antimonials; consideration of regional efficacy variability
Multidrug/general mechanisms [7]	Aneuploidy, gene amplification, and genomic plasticity	H-locus, MRPA, ABC, G6PDH	Increased copy number of resistance-related genes enabling rapid adaptation	Rational combination therapy; genomic and pharmacovigilance surveillance

Beyond these canonical pathways, emerging evidence highlights the role of P-glycoprotein-like ABC efflux pumps, gene amplification/deletion of key drug-target loci, and global metabolic rewiring as major drivers of clinical resistance. Efflux-mediated tolerance, particularly through overexpression of ABC family transporters, accelerates drug extrusion and reduces intracellular drug exposure, directly contributing to treatment failure. Likewise, structural alterations or copy-number variations in genes associated with antimonial and miltefosine susceptibility reshape drug–target interactions and impair drug uptake or activation. Metabolic remodeling—encompassing shifts in lipid biosynthesis, redox homeostasis, and mitochondrial function—further enhances parasite survival under sustained pharmacological pressure. Together, these concerted adaptations illustrate that resistance is not the result of a single mutation but arises from network-level plasticity, reinforcing the urgent need to identify nonredundant molecular vulnerabilities for next-generation therapeutics [171].

## 11. Conclusions

Leishmaniasis remains a major neglected tropical disease with complex clinical, immunological, and epidemiological dynamics that continue to challenge global health systems. Its pathogenesis reflects a finely tuned interplay between sand fly saliva, parasite virulence factors, host immune responses, and environmental determinants that shape the clinical spectrum of disease, from localized cutaneous lesions to severe mucocutaneous. Despite advances in understanding the molecular basis of host–parasite interactions, the persistence of the parasite within macrophages, its ability to evade immune responses, and the influence of comorbidities such as malnutrition and immunosuppression underscore the urgent need for integrated public health strategies.

Current therapeutic options, while effective in selected contexts, are increasingly compromised by toxicity and the emergence of molecular resistance mechanisms, including decreased drug uptake, upregulation of efflux transporters (such as MRPA), thiol-based detoxification pathways, and mitochondrial adaptations that reduce drug-induced oxidative stress. These processes collectively contribute to treatment failure and reinforce the urgent need to identify new therapeutic targets and host-directed strategies.

This reality highlights the importance of species identification, immune modulation approaches, and therapies that impair parasite survival pathways. Future perspectives include the development of drugs and vaccines targeting parasite virulence factors and metabolic vulnerabilities, as well as improved diagnostics enabling early and accurate species differentiation. The implementation of personalized treatment algorithms may further optimize therapeutic outcomes. Collectively, these advances have the potential to reduce disease burden and support more sustainable control—and eventual elimination—of leishmaniasis.

## 12. Future Aspects and Follow-Up Actions in Cutaneous and Mucocutaneous Leishmaniasis

Future efforts in cutaneous and mucocutaneous leishmaniasis should prioritize the development of safer, shorter, and species-specific treatment regimens, supported by robust clinical trials in endemic regions. Advances in point-of-care molecular diagnostics will be essential to improve species identification, predict therapeutic response, and detect early relapse. Research into host-directed therapies, including immunomodulatory agents and mitochondrial-targeted compounds, may enhance parasite clearance while reducing tissue damage. The integration of omic technologies—genomics, transcriptomics, and microbiome profiling—offers new opportunities to identify biomarkers of disease severity, treatment failure, and mucosal progression. Strengthening surveillance networks, ensuring pharmacovigilance, and expanding access to liposomal amphotericin B and miltefosine are critical for health systems. Long-term follow-up of treated patients is also necessary to monitor relapse, chronic sequelae, and mucosal involvement. Ultimately, combining improved diagnostics, precision therapies, and public health strategies will be key to reducing the global burden of CL and MCL.

## Data Availability

Not applicable.

## Abbreviations

The following abbreviations are used in this manuscript:

CL	Cutaneous leishmaniasis
MCL	Mucocutaneous leishmaniasis
VL	Visceral leishmaniasis
NTDs	Neglected tropical diseases
Th1/Th2	T helper 1/T helper 2 immune response
TLR	Toll-like receptor
NLR	NOD-like receptor
NK	Natural killer cell
DCs	Dendritic cells
ROS	Reactive oxygen species
LPG	Lipophosphoglycan
GIPLs	Glycosylinositol phospholipids
GP63	Glycoprotein 63 (leishmanolysin)
PPGs	Proteophosphoglycans
PSA-2	Parasite surface antigen-2
CR3	Complement receptor 3
NO	Nitric oxide
IFN-γ	Interferon-gamma
IL-10/IL-4/IL-12	Interleukins 10, 4, and 12
TNF-α	Tumor necrosis factor-alpha
SbV/SbIII	Pentavalent and trivalent antimony
HMGR	HMG-CoA reductase
ABC transporters	ATP-binding cassette transporters
AQP1	Aquaglyceroporin 1
PRRs	Pattern recognition receptors
APC	Antigen-presenting cell
GPI	Glycosylphosphatidylinositol
TSH2	Trypanothione
MAPK, NF-κB, STAT1	Intracellular signaling molecules
CCL2	Chemokine (C-C motif) ligand 2
LST	Leishmania skin test
MRPA	Multidrug Resistance Protein A
APX	Ascorbate Peroxidase
